# Clinicopathological Profile of a Cohort of Patients With Malignant Melanoma in the United Kingdom

**DOI:** 10.7759/cureus.39874

**Published:** 2023-06-02

**Authors:** Saroona Haroon, Iresha Vithanage, Khushbakht Rashid, Mahnoor Aslam, Heba Elmahdy, Shamail Zia, Umair Arshad Malik, Muhammad Irfan, Atif A Hashmi

**Affiliations:** 1 Pathology, King's Mill Hospital, Sutton-in-Ashfield, GBR; 2 Internal Medicine, Liaquat National Hospital and Medical College, Karachi, PAK; 3 Internal Medicine, Baqai Medical University, Karachi, PAK; 4 Public Health Sciences, University of Alberta, Edmonton, CAN; 5 Dermatology, King's Mill Hospital, Sutton-in-Ashfield, GBR; 6 Pathology, Jinnah Sindh Medical University, Karachi, PAK; 7 Internal Medicine, Aga Khan University, Karachi, PAK; 8 Statistics, Liaquat National Hospital and Medical College, Karachi, PAK; 9 Pathology, Liaquat National Hospital and Medical College, Karachi, PAK

**Keywords:** nodular melanoma, clarks level, breslow thickness, clinicopathological profile, malignant melanoma

## Abstract

Introduction

Malignant melanoma (MM) is potentially a fatal type of skin cancer and a major health concern for the Caucasian population. It is a heterogeneous disease with a wide spectrum of manifestations. Therefore, in this study, we evaluated the clinicopathological characteristics of MM.

Methods

We retrospectively studied the clinicopathological characteristics of MM in 167 biopsy-proven cases of MM reported between January 2020 and December 2021 at Kings Mill Hospital, Sutton-in-Ashfield, United Kingdom. Clinical data such as the age, sex, and anatomical site of the lesion were obtained from the clinical referral forms. Biopsies of the lesions were performed, and the specimens collected were sent to the laboratory for histopathological study and v-raf murine sarcoma viral oncogene homolog B1 (BRAF) mutation evaluation. Formalin-fixed paraffin-embedded blocks (FFPE) were prepared, sectioned, and stained with hematoxylin and eosin for histological examination.

Results

A total of 167 cases of MM were included in the study. The age range was 23-96 years, with the median age at diagnosis found to be 66 years; males were more commonly affected (52.1%). The median Breslow thickness was 1.20 mm. The median mitotic activity was 1.0/mm^2^. The lower limb was the most common site of involvement (27.5%), followed by the thorax (25.1%). The most common histological subtype was superficial spreading melanoma (SSM) (77.8%), followed by nodular melanoma (14.4%). The in situ component was present in 95.8% of cases; a majority (92.2%) of the cases showed vertical growth phase, 71.9% of cases were at Clark’s level IV of invasion, regression was noted in 70.7% of cases, ulceration was present in 21.6% of cases, and microsatellites were present in 3% of cases. Perineural invasion was present in 3% of cases, and lymphovascular invasion (LVI) was present in 4.2% of cases. BRAF mutation testing was performed on 36 cases, out of which 20 cases (55.6%) showed BRAF mutation. Acral lentiginous melanoma and nodular melanoma were most likely to show ulceration (66.7% and 37.5%, respectively). SSM and lentigo maligna melanoma were more likely to be associated with regression.

Conclusion

The study demonstrated that MM is prevalent among the elderly population with male predominance; SSM was found to be the most common subtype. The study further demonstrated various clinicopathological features of MM and its association with histological subtypes.

## Introduction

Malignant melanoma (MM) is a neoplasm of melanocytic cells and most commonly affects the skin. However, it may involve mucosal surfaces such as the oral cavity, uveal tract of the eye, gastrointestinal mucosa, and leptomeninges [1]. It is the most aggressive type of skin cancer with a high mortality rate, accounting for more than 70% of deaths from all skin cancers [2]. It is estimated that by 2040, MM will be the second most common cancer in the United States [3]. Although a recent study suggests that the mortality rate of MM of the skin has declined in recent years, the incidence of MM of the skin continues to increase by 2.2% per year [4]. It accounts for 1.7% of all cancer burden globally, with approximately 325,000 new cases estimated to be reported in 2020 [5]. A marked geographical variation has been reported in the incidence and mortality rate of MM, with Australia and New Zealand reporting the highest number of cases. Caucasians and fair-skinned males are more susceptible to developing MM [6]. It is a heterogeneous tumor, and based on the growth pattern, cutaneous melanoma or MM has been classified into four major sub-types; superficial spreading melanoma (SSM), which is the most common type, affecting the trunk and the extremities, lentigo maligna melanoma (LMM), which most commonly affect the sun-exposed areas in elderly individuals, having irregular margins, acral lentiginous melanoma (ALM) affecting hands and feet, and nodular melanoma (NM), which shows deep pigmentation and grows rapidly [7].

MMs have two phases of growth: a horizontal (radial) growth phase that only spreads through the epidermis and a vertical phase that extends into the dermis [6]. MM is a multifactorial tumor caused by a combination of environmental and genetic risk factors. A major environmental risk factor of MM is ultraviolet radiation from both solar and artificial sources, and it is estimated to be the cause of MM in 50-90% of cases [8]. The important risk factors related to the host are skin phototype (phototype I and II at high risk), pre-existing melanocytic nevi, genetic susceptibility, and family history such as familial MM [9]. Staging of MM is of crucial importance to determine the prognosis of the disease, and for the start of appropriate treatment, the American Joint Committee on Cancer (AJCC) revised the tumor, node metastasis (TNM) staging guidelines on MM, which include Breslow thickness, ulceration, and mitotic rate. These three features are considered to be the most important factors for prognosis and staging [10].

MM displays a wide spectrum of clinicohistological manifestations. Hence, the identification and reporting of the disease is extremely important for the commencement of the specific treatment for better outcomes. Therefore, demographic details, clinical characteristics, and diagnostic measures are essential not only for histopathological reports but also for implying significant clinical concern in treatment options, which can only be achieved by regular organized studies. Therefore, this study aimed to determine the clinicopathological profile of MM and its clinical correlation in a United Kingdom (UK) population.

## Materials and methods

We conducted a retrospective cross-sectional study at Kings Mill Hospital, Sutton-in-Ashfield, UK. A total of 167 cases of MM that were reported between January 2020 and December 2021 at our institute were enrolled in the study. All biopsy-proven cases of MM were included in the study. Clinicopathological data of the cases included in the study reported during the study period were retrieved from the institutional archive. Clinical data were collected from the clinical referral forms, which included the patient’s age, gender, and the site of the lesion. Cases with missing clinical data or tissue block and benign cases were excluded from the study. Institutional Review Board approval was not needed as it was a retrospective study. The clinical audit project was approved by Sherwood Forest Hospitals NHS Foundation Trust (Project Code: Path/CA/2021-22/04).

Out of 167 cases, excisional biopsy was performed in 156, punch biopsy was performed in six, incisional biopsy was performed in three, and specimens were collected by the curette and shaving method in two cases. All specimens obtained were fixed with 10% neutralized formalin and were sent to the laboratory for histopathological evaluation. For histological examination, tissue blocks were prepared by removing water from the tissue by alcohol dehydration, which were then treated with xylene to clear off the alcohol from the tissue sample and then embedded in the paraffin wax at 56°C, after which they were cut into 4-5 µm sections. The sections are placed on a slide treated with L-lysine, dried, and sequentially treated with xylene, alcohol, and water, and were subsequently stained with hematoxylin and eosin, and were studied under a microscope by a senior histopathologist. The histological subtype, melanoma in situ, microsatellite lesions, ulceration, regression, tumor-infiltrating lymphocytes (TIL), growth phase, perineural invasion (PNI), lymphovascular invasion (LVI), and Clark’s level of invasion were observed. Breslow thickness, i.e., the distance between the granular layer of the epithelium and the deepest part of the tumor was measured using an ocular micrometer, and the mitotic rate was estimated by calculating the number of mitotic figures in the tumor cells within 10 high-power fields (HPF).

The v-raf murine sarcoma viral oncogene homolog B1 (BRAF) mutation was detected by the amplification-refractory mutation system (ARMS)-polymerase chain reaction (PCR) method. The 4-5 µm thick sections of formalin-fixed paraffin-embedded (FFPE) tissue were deparaffinized and treated with proteinase K at 56°C for digestion. Using the ZR Genomic DNA™ I kit (Zymo Research Corporation, Irvine, California, United States), genomic DNA extraction was performed as per the guidelines. Quantification of the extracted DNA was performed using a NanoDrop 1000 Spectrophotometer V3.8 (Thermo Fisher Scientific, Waltham, Massachusetts, United States). PCR was performed in a final volume of 25 μl containing 1x buffer, 2 mM magnesium chloride (MgCl2), 400 nM primer FO (forward outer), 200 nM primer RO (reverse outer), and Fiwt, 800 nM primer Rimut, HotStarTaq DNA Polymerase (QIAGEN, Hilden, Germany) 1 unit, 200 μM each deoxynucleoside triphosphate (dNTP), and 30 ng genomic DNA template. PCR amplification was performed. The product of PCR was analyzed with 2% agarose gel.

Data analysis

Data analysis was performed using the IBM SPSS Statistics for Windows, Version 26.0 (Released 2019; IBM Corp., Armonk, New York, United States). The median for age, Breslow thickness, mitotic activity, peripheral margins, and deep margins were calculated, whereas frequencies and percentages for all other clinicopathological variables were evaluated. A p-value of < 0.05 was considered significant. Fisher’s exact tests were applied to determine the association of various clinicopathological features concerning the histological subtypes.

## Results

A total of 167 cases of MM were included in the study. The median age in our study was 66±23 years (Table 1). MM was found to be prevalent in elderly patients above 65 years of age (50.9%). The median Breslow thickness was found to be 1.2 mm. The median mitotic activity was found to be 1.0/mm². The median peripheral margin of the excised lesions was found to be 3 mm and the deep margins were 4 mm. Males were most affected (52.1%) than females (47.9%). The most common site of occurrence was the lower limb (27.5%), followed by the thorax (25.1%), upper limb (21%), and head and neck (17.4%). Most cases gave the clinical impression of MM (74.3%), whereas few lesions gave the clinical impression of dysplastic nevus (10.8%) and squamous cell carcinoma (6.6%). In 156 cases (93.4%), excisional biopsy was performed, whereas in six cases punch biopsy (3.6%) was performed, in three cases specimen was acquired by incisional biopsy, and in one case, specimen was obtained by curette biopsy. The orientation of the specimen was given in 44.3% of cases. In 95.8% of cases, the in situ component was present. In 92.2% of cases, a vertical growth phase was observed, whereas 7.8% showed a radical growth phase. Of the cases, 71.9% were at Clark level IV, 13.8% were at Clark level III, 10.2% were at Clark level V, and 7% were at Clark level II. Ulceration was noted in 21.6% of cases, microsatellite lesions were present in 3% of cases, and regression was observed in 70.7% of cases, TILs were present in 19.2% of cases, LVI was present in 4.2% of cases, and PNI was present in 3% of cases. BRAF mutation testing was performed on 36 cases, out of which 20 cases (55.6%) showed BRAF mutation. The most common histological type was found to be SSM, diagnosed in 77.8% of cases; the second most common type among our patients was found to be NM (14.4%), followed by LMM (6%) and ALM (1.8%), as shown in Table 1.

**Table 1:** Clinicopathological profile of study population IQR:  interquartile range; BRAF: v-raf murine sarcoma viral oncogene homolog B1

Clinicopathological parameters	Values
Age (years), median (IQR)	66.0 (23.0)
Age groups	
21-35 years, n (%)	9 (5.4)
36-50 years, n (%)	26 (15.6)
51-65 years, n (%)	47 (28.1)
>65 years, n (%)	85 (50.9)
Breslow thickness (mm), median (IQR)	1.20 (2.70)
Mitotic activity/mm^2^, median (IQR)	1.0 (5.0)
Peripheral margin (mm), median (IQR)	3.0 (3.0)
Deep margin (mm), median (IQR)	4.0 (3.60)
Gender	
Male, n (%)	87 (52.1)
Female, n (%)	80 (47.9)
Anatomical site	
Head and neck, n (%)	29 (17.4)
Thorax, n (%)	42 (25.1)
Abdomen, n (%)	15 (9)
Upper limb, n (%)	35 (21)
Lower limb, n (%)	46 (27.5)
Clinical impression	
Malignant melanoma, n (%)	124 (74.3)
Dysplastic nevus, n (%)	18 (10.8)
Lentigo maligna,n (%)	1 (0.6)
Squamous cell carcinoma, n (%)	11 (6.6)
Hemangioma, n (%)	4 (2.4)
Basal cell carcinoma, n (%)	3 (1.8)
Seborrheic keratosis, n (%)	4 (2.4)
Nevus, n (%)	1 (0.6)
Ulcer, n (%)	1 (0.6)
Specimen type	
Excision, n (%)	156 (93.4)
Punch, n (%)	6 (3.6)
Incisional, n (%)	3 (1.8)
Curette, n (%)	1 (0.6)
Shave, n (%)	1 (0.6)
Orientation	
Yes, n (%)	74 (44.3)
No, n (%)	93 (55.7)
In situ component	
Present, n (%)	160 (95.8)
Absent, n (%)	7 (4.2)
Growth phase	
Vertical, n (%)	154 (92.2)
Radial, n (%)	13 (7.8)
Clark’s level	
Level II, n (%)	7 (4.2)
Level III, n (%)	23 (13.8)
Level IV, n (%)	120 (71.9)
Level V, n (%)	17 (10.2)
Ulceration	
Present, n (%)	36 (21.6)
Absent, n (%)	131 (78.4)
Microsatellites	
Present, n (%)	5 (3)
Absent, n (%)	162 (97)
Regression	
Present, n (%)	118 (70.7)
Absent, n (%)	49 (29.3)
Tumor infiltrating lymphocytes	
Brisk, n (%)	32 (19.2)
Non-brisk, n (%)	135 (80.8)
Lymphovascular invasion	
Present, n (%)	7 (4.2)
Absent, n (%)	160 (95.8)
Perineural invasion	
Present, n (%)	5 (3)
Absent, n (%)	162 (97)
BRAF mutation (n=36)	
Present, n (%)	20 (55.6)
Absent, n (%)	16 (44.4)
Histological subtype	
Superficial spreading, n (%)	130 (77.8)
Lentigo maligna, n (%)	10 (6)
Nodular, n (%)	24 (14.4)
Acral lentiginous, n (%)	3 (1.8)

Table 2 demonstrates the relationship between various clinicopathological factors and the histological subtypes of MM. A significant association of histological subtype of MM was noted with age, anatomical site, presence of in situ component and ulceration, and regression. We found that SSM, LMM, and NM were more prevalent in elderly patients > 65 years of age (43.8%, 90%, and 75%, respectively) whereas ALM was found to affect patients between 51 and 65 years of age. SSM and ALM most commonly affected female patients (50.8% and 66.7%, respectively), whereas LMM and NM predominantly affected males (80% and 58.3%, respectively), but the difference is not statistically significant. SSM and ALM most commonly affected the lower limbs (29.2% and 100%, respectively), LMM most commonly involved the head and neck region (90%), and NM mostly occurred on the upper limb (33.3%) with a statistically significant difference. The presence of in situ lesions showed a significant association with SSM, LMM, and ALM subtypes as they were present in all the cases of SSM, LMM, and ALM compared with NM (70.8%). ALM and NM were significantly more likely to possess ulceration, present in 66.7% and 37.5% of cases, respectively, compared with SSM and LMM in which ulceration was noted in 17.7% and 20% of cases, respectively. SSM, LMM, and NM significantly showed regression (73.8%, 80%, and 58.3%, respectively) compared with ALM, which showed no regression in any case. No significant association of MM subtype was noted with the growth phase. All subtypes in most cases did not show PNI or LVI. Microsatellites were not present in most of the cases of each subtype. All subtypes mostly demonstrated Clark’s level IV tumor invasion. BRAF mutation evaluation was evaluated in 36 cases and 62.5% of NM cases showed BRAF mutation, while it was seen in 54.2% cases of SSM and 50% each of ALM and LMM; however, the association was not statistically significant.

**Table 2:** Association of clinicopathological parameters with histological type of malignant melanoma BRAF: v-raf murine sarcoma viral oncogene homolog B1 *Fisher exact test was applied, **p-value significant as <0.05

Clinicopathological parameters	Histological subtype of melanoma				p-value
	Superficial spreading	Lentigo maligna	Nodular	Acral lentigenous	
Age group*					
21-35 years, n (%)	9 (6.9)	0 (0)	0 (0)	0 (0)	0.039**
36-50 years, n (%)	23 (17.7)	1 (10)	2 (8.3)	0 (0)	
51-65 years, n (%)	41 (31.5)	0 (0)	4 (16.7)	2 (66.7)	
>65 years, n (%)	57 (43.8)	9 (90)	18 (75)	1 (33.3)	
Gender					
Male, n (%)	64 (49.2)	8 (80)	14 (58.3)	1 (33.3)	0.224
Female, n (%)	66 (50.8)	2 (20)	10 (41.7)	2 (66.7)	
Anatomical site*					
Head and neck, n (%)	16 (12.3)	9 (90)	4 (16.7)	0 (0)	<0.001**
Thorax, n (%)	37 (28.5)	0 (0)	5 (20.8)	0 (0)	
Abdomen, n (%)	12 (9.2)	1 (10)	2 (8.3)	0 (0)	
Upper limb, n (%)	27 (20.8)	0 (0)	8 (33.3)	0 (0)	
Lower limb, n (%)	38 (29.2)	0 (0)	5 (20.8)	3 (100)	
In situ component*					
Present, n (%)	130 (100)	10 (100)	17 (70.8)	3 (100)	<0.001**
Absent, n (%)	0 (0)	0 (0)	7 (29.2)	0 (0)	
Growth phase*					
Vertical, n (%)	119 (91.5)	8 (80)	24 (100)	3 (100)	0.166
Radial, n (%)	11 (8.5)	2 (20)	0 (0)	0 (0)	
Clark’s level*					
Level II, n (%)	6 (4.6)	1 (10)	0 (0)	0 (0)	0.373
Level III, n (%)	20 (15.4)	2 (20)	1 (4.2)	0 (0)	
Level IV, n (%)	92 (70.8)	7 (70)	19 (79.2)	2 (66.7)	
Level V, n (%)	12 (9.2)	0 (0)	4 (16.7)	1 (33.3)	
Ulceration*					
Present, n (%)	23 (17.7)	2 (20)	9 (37.5)	2 (66.7)	0.037**
Absent, n (%)	107 (82.3)	8 (80)	15 (62.5)	1 (33.3)	
Microsatellites*					
Present, n (%)	3 (2.3)	0 (0)	2 (8.3)	0 (0)	0.340
Absent, n (%)	127 (97.7)	10 (100)	22 (91.7)	3 (100)	
Regression*					
Present, n (%)	96 (73.8)	8 (80)	14 (58.3)	0 (0)	0.022**
Absent, n (%)	34 (26.2)	2 (20)	10 (41.7)	3 (100)	
Tumor infiltrating lymphocytes*					
Brisk, n (%)	29 (22.3)	2 (20)	1 (4.2)	0 (0)	0.158
Non-brisk, n (%)	101 (77.7)	8 (80)	23 (95.8)	3 (100)	
Lymphovascular invasion*					
Present, n (%)	4 (3.1)	0 (0)	3 (12.5)	0 (0)	0.230
Absent, n (%)	126 (96.9)	10 (100)	21 (87.5)	3 (100)	
Perineural invasion*					
Present, n (%)	3 (2.3)	0 (0)	2 (8.3)	0 (0)	0.340
Absent, n (%)	127 (97.7)	10 (100)	22 (91.7)	3 (100)	
BRAF mutation (n=36)*					
Present, n (%)	13 (54.2)	1 (50)	5 (62.5)	1 (50)	1.000
Absent, n (%)	11 (45.8)	1 (50)	3 (37.5)	1 (50)	

## Discussion

This study was conducted to evaluate the clinicopathological profile of MM in a cohort of patients in the UK. We concluded that MM was most prevalent in the elderly population of greater than 65 years of age with male predominance. The lower limb was found to be the most common site of occurrence. The most common histological subtype was found to be SSM (77.8%), with NM (14.4%) being the second most common subtype. In our study, most cases (92.2%) demonstrated a radical growth phase, with most cases at Clark level IV of invasion, and the median mitotic activity was 1.0 mm². In most cases, an in situ lesion was present. Regression was noted in most cases (70.3%). Ulceration, microsatellite lesion, and TIL showed less frequency in our cases. Similarly, PNI and LVI were less frequently noted in our study.

Panda et al. conducted a study on clinicopathological characteristics of MM in 182 cases, and similar to our study, they corroborated that MM most commonly occurs in the sixth decade of life, with males predominating [11]. In most cases, the lower limb was the most common site of involvement; similarly, most cases showed Clark’s level IV invasion. Unlike our study, they found ALM (59.4%) to be the most common subtype diagnosed, followed by SSM (32.9%). In concordance with our study, various other studies demonstrated that MM affected older patients, predominantly males of greater than 65 years of age, with lower extremities being the predominant site of involvement [12-14]. Similar to our finding of SSM being the most common subtype, Baykal et al. conducted a study in Turkey and found SSM to be the most common subtype (37.19%) in their study [15], whereas various studies conducted previously in different regions found ALM to be the predominate subtype [16,17].

Breslow thickness of the tumor is an important prognostic factor; tumors of less than 0.76 mm have the least risk for metastasis, whereas tumors 1.5 mm thick are at high risk for metastasis. Similarly, ulceration is the second most important prognostic factor; tumors with increased thickness are more likely to be ulcerated and are associated with a worse prognosis. The mitotic rate is an important predictor of survival when analyzed in the vertical growth phase [10]. In our study, the median Breslow thickness was 1.2 mm. Gulliver et al. found that most cases in their study had a Breslow thickness of 1.0 mm [14].

Furthermore, we found that SSM and ALM most commonly involved the lower limb, whereas LMM mostly occurred on the head and neck region and NM mostly involved the upper limb. The in situ lesion was more likely to be associated with SSM, LMM, and ALM. Ulceration was more likely to occur in cases of ALM and NM, and regression was noted in cases of SSM, LMM, and NM. PNI and LVI were more likely to be present in cases of NM, and TIL was found to be more likely to be present in cases of SSM. A study was conducted by El Sharouni et al. on 48,361 cases of MM [18]; similar to our study, they found SSM to be the most common subtype (79.3%) followed by NM, and contrary to our study, their study demonstrated female predominance. The median Breslow thickness in their study was relatively less, 0.8 mm, ulceration was present in 12.5% of cases, the trunk was the most common site of involvement in the majority of the cases (42.3%), SSM and NM mostly involved the trunk, and most LMM were located on the face and most ALM on the feet, whereas in our study SSM, ALM, and NM predominantly involved the lower limb, LMM involved the head and neck region. Ulceration was mostly present in cases of NM (38.7%) followed by ALM (34.45%). Contrarily, in our study, ALM (66.7%) showed ulceration in most cases followed by NM. The presence of TIL in a tumor is a valuable marker for immune response and has a favorable prognostic value; in a study conducted previously by Lin et al. [19]., they found fewer TILs in NM, indicating the aggressive nature of NM. In our study, SSM seemed more likely to have TIL, although no statistically significant correlation could be found between TIL and the histological subtype. Tas et al. conducted a study on 1017 cases of MM and concluded that NM and ALM are associated with clinicopathological factors associated with poor prognosis such as higher Clark level of invasion, thick Breslow depth, presence of ulceration, and LVI [20]. Farahmand et al. studied the histopathological findings and tumor stage in various types of MM, and similar to our study, they found that NM and ALM showed the highest rates of metastasis, microsatellites, PNI, and Clark level of invasion [21]. In our study, all subtypes of MM in most cases were at Clark’s level IV, whereas a study conducted by Vilanova et al. on the histopathological profile of cutaneous MM in Brazil on 313 patients found a correlation between histological subtypes and Clark’s level of invasion, and that majority of the cases of NM and ALM were at a higher level of invasion (NM at Clark level IV or V and ALM at Clark level V), and that LMM and SMM were more likely to present at a lower level of invasion (LMM predominantly at Clark level II and SSM at Clark level III) [22].

BRAF mutations are reported to be present in approximately 50% of the cases of MM and are of therapeutic importance as they show a positive response to treatment with BRAF inhibitors [23]. In our study, BRAF mutation was found to be present in 55.6% of cases; however, we performed BRAF testing in only 36 cases. 

Molecular/gene expression profiling has provided new insights into the understanding of MM. Based on genomic profiling, four distinct genomic subtypes of MM are identified, these include BRAF-mutant, NF1-loss, NRAS-mutant, and triple wild-type (TWT). The later subtype (TWT) lacks the characteristic BRAF, RAS, and NF1 molecular alterations. It has also been shown that these molecular subtypes don’t correspond to histological subtypes or locations; however, acral and mucosal melanomas are more likely to be TWT [24]. However, in our study, we didn’t evaluate molecular alteration except for BRAF in a limited number of cases, and we didn’t find any significant association of BRAF mutation with MM histological subtype.

Limitations

The study had a few limitations. The study was conducted in a single center and with a limited sample size. Moreover, as this was a retrospective study, follow-up of the patient was not determined. Also, risk factor stratification and therapeutic intervention studies were not conducted. Therefore, we propose that further prospective multicenter studies be conducted to understand the correlation of various clinicopathological characteristics with different subtypes of MM. Moreover, apart from BRAF, molecular testing for RAS and NF1 was not conducted in our study and BRAF was tested in a limited sample. 

## Conclusions

In accordance with the studies done previously on MM, we found that MM was more prevalent among the elderly population and the risk of developing MM increases with increasing age, with male predominance. SSM was found to be the most common subtype. The lower limb was the most common site of occurrence. Moreover, we found that the SSM and LMM were more likely to be associated with favorable histological features (regression), whereas NM and ALM showed an association with poor histological parameters (ulceration).
